# Supplementary material for: Relationship between Tumor Mutational Burden, PD-L1, Patient Characteristics, and Response to Immune Checkpoint Inhibitors in Head and Neck Squamous Cell Carcinoma
Source: Cancers (Basel). 2021 Nov 16;13(22):5733. doi: 10.3390/cancers13225733 (PMC8616373; doi:10.3390/cancers13225733)
Supplement: Supplementary file 1 [file cancers-13-05733-s001.zip › cancers-1413798-supplementary.pdf]

Table S1. Characterization of Patients who Received Immunotherapy.

Patient Number	ICI	Number of Treatments	Other Treatments Concurrent with ICI	Best OR	PFS (Days)	TMB (mut/Mb)	PD-L1 (%)	TPS vs CPS
1	P	7	RT	PD	132	7	1	TPS
2	P	5	-	PD	105	5	60	TPS
3	N	5	-	PD	106	4	0	TPS
4	P	23	CT	CR	477	1	2	CPS
5	P	3	-	CR	1875	11	70	TPS
6	P	5	-	PD	105	9	50	TPS
7	P	34	-	CR	880	10	20	TPS
8	P	5	-	PD	105	0	70	TPS
9	D	3	Vaccine Protocol	PD	105	13	10	TPS
10	P	30	-	PR	477	4	0	CPS
11	P	17	-	PR	445	3	--	--
12	P	12	-	PR	252	5	60	TPS
13	N	4	IO Protocol	PD	84	3	--	--
14	P	5	-	PD	105	9	1	TPS
15	P	17	-	CR	750	35	5	TPS
16	P	35	RT	CR	970	11	5	TPS
17	P	5	-	PD	105	3	1	TPS
18	P	35	CRT	CR	1760	5	--	--
19	P	20	-	PR	465	8	70	CPS
20	P	5	-	PD	105	4	--	--
21	P	5	-	PD	105	5	--	--
22	P	7	-	PD	147	3	0	TPS
23	P	35	-	CR	1000	28	1	TPS
24	P	4	-	PD	84	1	1	TPS
25	P	35	RT	CR	850	10	10	CPS
26	P	5	-	PD	105	5	60	TPS
27	P	35	-	CR	860	10	30	CPS
28	P	12	RT	SD	252	4	10	CPS
29	P	7	-	SD	185	16	0	CPS
30	P	18	-	CR	670	33	100	CPS
31	P	13	-	SD	273	--	2	CPS
32	P	6	-	PD	126	--	15	CPS
33	P	7	-	PD	147	4	90	CPS
34	P	5	-	PD	105	--	--	--
35	P	5	-	PD	105	1	1	2
36	P	5	-	PD	105	--	--	--
37	P	4	-	PD	84	--	--	--
38	N	7	-	PD	98	3	--	--
39	N	9	-	PD	126	6	--	--
40	P	5	-	PD	105	--	40	CPS
41	P	30	-	PR	670	16	1	CPS
42	P	13	-	PR	270	0	5	CPS
43	P	9	-	PD	190	4	10	CPS
44	N	8	IO Protocol	PD	112	--	--	--
45	P	5	-	PD	105	9	40	TPS

46	P	11	-	PR	232	5	10	CPS
47	P	18	-	PR	375	--	--	--
48	D	2	Vaccine Protocol	PD	63	--	--	--
49	P	18	RT	CR	334	11	100	CPS
50	P	15	-	PR	321	--	N.A	--
51	P	18	-	PR	370	--	--	--

CPS, combined positive score; CR, complete response; CT, chemotherapy; D, Durvalumab; ICI, immune checkpoint inhibitor; IO, immunotherapy; N, Nivolumab; OR, overall response; P, Pembrolizumab; PD, progressive disease; PD-L1, Program Death-Ligand1; PFS, progression free survival; PR, partial response; RT, radiotherapy; SD, stable disease; TMB, tumor mutational burden; TPS, tumor proportion score, “-,“ none; “--," not applicable.
